# Microlancet-assisted internal urethrotomy with stent placement for feline pelvic urethral stricture: a case report

**DOI:** 10.3389/fvets.2026.1755771

**Published:** 2026-03-25

**Authors:** Danfu Ma, Wijit Sutthiprapa, Xinyi Xu, Yanbing Zhao, Wannasit Chantornvong

**Affiliations:** 1 College of Veterinary Medicine, Nanjing Agricultural University, Nanjing, China; 2 Veterinary Teaching Hospital, Nanjing Agricultural University, Nanjing, China; 3 Veterinary Teaching Hospital, Kasetsart University, Bangkok, Thailand; 4 Parichart Suwinthawong Animal Hospital, Bangkok, Thailand

**Keywords:** cat, fluoroscopy, internal urethrotomy, microlancet, minimally invasive, nitinol stent, urethral stricture

## Abstract

**Background:**

Intrapelvic urethral strictures in male cats are challenging to manage. Standard perineal urethrostomy cannot address lesions within the pelvic canal, and alternative open surgical techniques carry high morbidity.

**Case description:**

A 5-year-old intact male domestic shorthair cat with concurrent hypertrophic cardiomyopathy and polycystic kidney disease presented with a 7-day history of stranguria. Diagnostic imaging revealed a focal 1-mm stricture in the post-prostatic urethra.

**Procedure:**

Under fluoroscopic guidance, retrograde internal urethrotomy was performed using a 1.3 × 130 mm urethral microlancet via a dorsal approach. A self-expanding laser-cut nitinol stent (5.0 mm × 50 mm) was deployed across the stricture. A double-lumen balloon catheter was placed through the stent for 14 days to promote mucosal integration and prevent migration.

**Results:**

The procedure was completed in 30 min with no complications. Urethral patency was immediately restored. Follow-up cystoscopy at 7 and 14 days demonstrated progressive epithelial ingrowth and near-complete encapsulation of the stent without restenosis. The cat regained normal urination.

**Conclusion:**

This report describes the first successful use of microlancet internal urethrotomy combined with nitinol stenting for an intrapelvic urethral stricture in a cat. The technique offers a promising minimally invasive alternative to conventional urethrostomy in high-risk cases.

## 1 Introduction

Urethral obstruction in male cats is a common clinical emergency often resulting from urethral plugs, urolithiasis, or idiopathic inflammation. In chronic or recurrent cases, fibrosis may lead to urethral stricture formation. While the penile urethra is the most frequent site of obstruction, lesions occurring within the intrapelvic urethra present a greater therapeutic challenge due to limited surgical access and poorer visualization.

Perineal urethrostomy (PU) is the most commonly used surgical solution for distal urethral obstruction in male cats (1), redirecting urinary flow through a new stoma at the level of the wide post-prostatic urethra. However, this approach does not resolve strictures situated proximally within the pelvic canal. Antepubic urethrostomy, while anatomically suitable, is associated with severe complications including urine scalding, incontinence, and dehiscence (2). In recent years, fluoroscopic-guided techniques such as balloon dilation and metallic stenting have emerged as alternatives in both veterinary and human patients, though evidence in feline medicine remains limited (1, 3–6).

Internal urethrotomy using a microlancet is a well-established method in human urology for the management of short urethral strictures (7), often performed under endoscopic guidance with adjunctive stenting to maintain lumen patency. Adaptation of this technique to feline patients requires bespoke instrumentation due to their narrow urethral diameter. This case explores the procedural feasibility, clinical rationale, and the translational potential of human-derived interventional techniques in small animal urology. In human urology, microlancet urethrotomy for short-segment strictures (<2 cm) achieves initial success rates of 70–85%, though recurrence is common without adjunctive stenting (8, 9). Here, we report the first documented use of a microlancet urethrotome combined with nitinol stenting for a post-prostatic urethral stricture in a cat, supplemented by a 14 Fr double-lumen catheter (inflated) to support stent integration and reduce migration risk. We deflated the double-lumen catheter, and withdrawn from the penile urethra 14 days later to keep reduce migration risk postoperatively.

## 2 Case description

### 2.1 Signalment and history

A 5-year-old, 4.9 kg, intact male domestic shorthair cat presented to the Veterinary Teaching Hospital of Nanjing Agricultural University with a 1-week history of stranguria, pollakiuria, and anorexia. The cat was previously healthy, housed indoors, and had no known exposure to toxins or urolith-forming diets.

### 2.2 Physical examination and comorbidities

On physical examination, the cat was quiet but responsive, estimated to be 5% dehydrated, with normal heart and respiratory rates. Abdominal palpation revealed renomegaly and an irregular contour of the left kidney. No urethral pulsation or obvious obstruction could be palpated caudally.

### 2.3 Clinicopathological findings

A complete blood count was unremarkable as shown in Table 1. Serum biochemistry revealed hyperglycemia (glucose 10.63 mmol/L, RI 4.11–8.84) and elevated creatine phosphokinase (CPK 741 U/L, RI 50–450), with no azotemia, electrolyte disturbances, or hepatic enzyme abnormalities.

**Table 1:** Hematology and biochemistry results before operation.

Parameter	Result	Reference interval	Unit
Total protein	62.7	54–89	g/L
Albumin	29.5	22–45	g/L
Globulin	33.2	15–57	g/L
Total bilirubin	2.44	2–15	μmol/L
Alanine aminotransferase (ALT)	44	8.2–123	U/L
Alkaline phosphatase (ALP)	13	10–90	U/L
Blood urea nitrogen (BUN)	7.20	3.6–15.5	mmol/L
Creatinine	121	27–223	μmol/L
Creatine phosphokinase (CPK)	741*	50–450	U/L
Glucose	10.63*	4.11–8.84	mmol/L
Cholesterol	2.10	1.68–5.81	mmol/L
Calcium	2.38	1.95–2.95	mmol/L
Phosphorus (inorganic)	1.65	1–2.74	mmol/L
White blood cells (WBC)	13.25	2.87–17.02	×10^9^/L
Neutrophils	10.43	2.30–10.29	×10^9^/L
Lymphocytes	1.80	0.92–6.88	×10^9^/L
Monocytes	0.78	0.05–0.67	×10^9^/L
Red blood cells (RBC)	6.23	6.54–12.20	×10^12^/L
Hemoglobin (HGB)	102	98–162	g/L
Hematocrit (HCT)	28.5	30.3–52.3	%
Mean corpuscular volume (MCV)	45.8	35.9–53.1	fL
Mean corpuscular hemoglobin (MCH)	16.4	11.8–17.3	pg
Mean corpuscular Hb concentration (MCHC)	358	281–358	g/L
Platelets (PLT)	304	151–600	×10^9^/L

*Indicating the parameter is abnormal.

**Figure 1:**
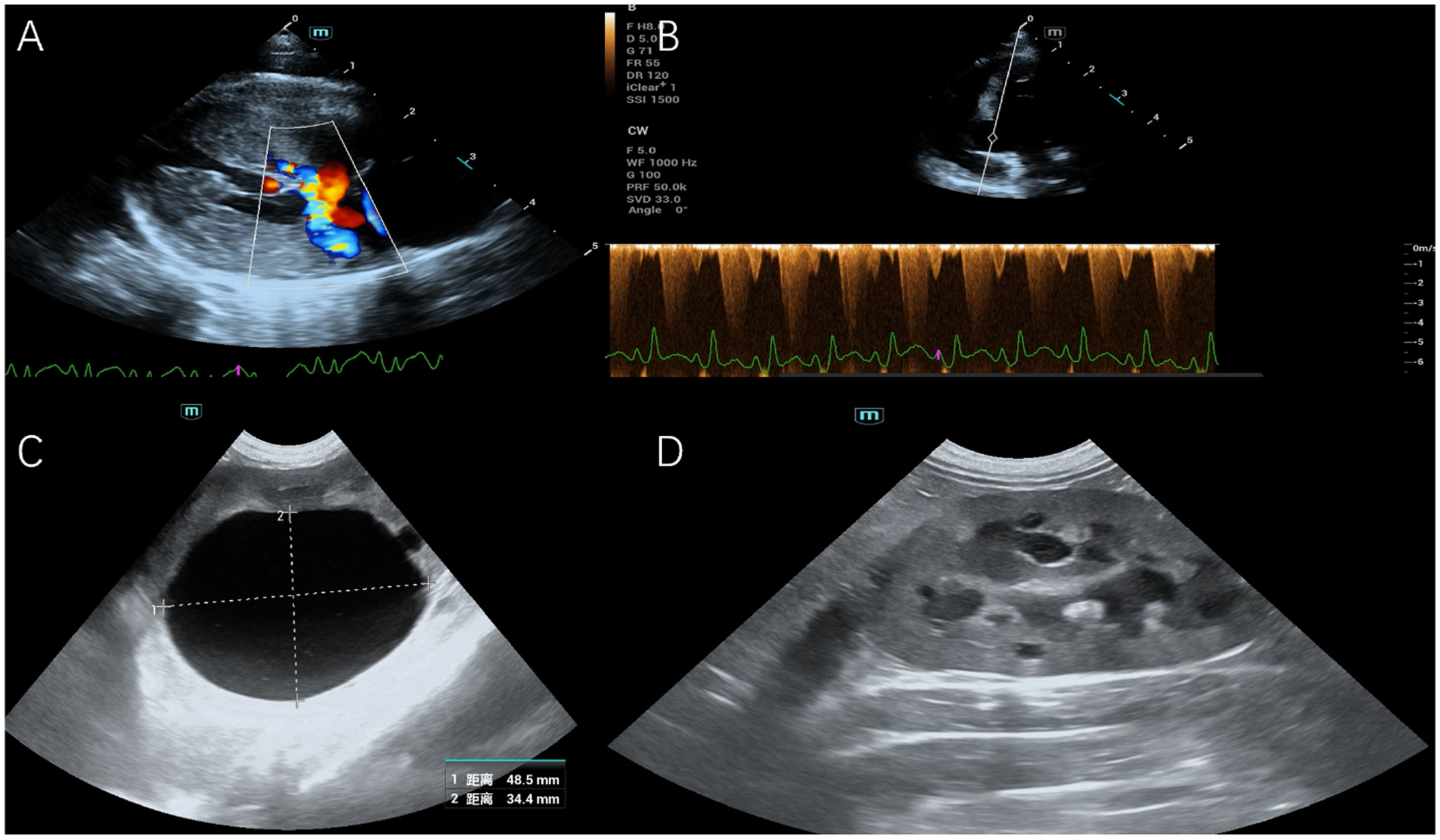
**(A)** Right parasternal long-axis echocardiographic view demonstrating concentric left ventricular hypertrophy and associated mitral regurgitation. **(B)** Apical 5-chamber echocardiographic view with color Doppler, revealing high-velocity flow (5.3 m/s) in the left ventricular outflow tract, consistent with dynamic obstruction. **(C)** Abdominal ultrasonogram of the left kidney, showing renomegaly with numerous cortical cysts and a large (48.5 × 34.4 mm) pelvic cyst. **(D)** Abdominal ultrasonogram of the right kidney, demonstrating renomegaly and diffuse multifocal cortical cysts.

Echocardiographic evaluation showed moderate concentric hypertrophy of the interventricular septum (IVS) and left ventricular posterior wall (LVPW), systolic anterior motion (SAM) of the mitral valve, mitral regurgitation, and a left ventricular outflow tract (LVOT) velocity of 5.3 m/s, as shown in Figures 1A,B. These findings were consistent with a hypertrophic cardiomyopathy (HCM) phenotype with dynamic LVOT obstruction. There was no evidence of systemic hypertension or hyperthyroidism.

Abdominal ultrasonography identified bilateral renomegaly, with the left kidney measuring 55 mm and the right 47 mm in length, as shown in Figures 1C,D. Both kidneys displayed multiple small anechoic cortical cysts, with a markedly distended left renal pelvis containing a single 5.0 cm anechoic cyst. The urinary bladder was moderately distended without intraluminal debris. Given the discrepancy between imaging findings and the severity of clinical signs, further investigation into the urethral patency was undertaken.

### 2.4 Clinicopathological findings

This study was approved by the Institutional Animal Welfare and Ethics Committee (or Ethics Committee for Animal Experimentation) of Nanjing Agricultural University, with approval reference number NJAULLSC2025104.

## 3 Treatment and outcome

### 3.1 Anesthesia

The cat was induced with intravenous propofol (4 mg/kg to effect). Anesthesia was maintained with isoflurane in oxygen using a Dräger Fabius Plus. The cat was placed in oblique lateral recumbency with the upper hindlimb secured cranially. End-tidal CO₂, SpO₂, ECG, temperature, and non-invasive blood pressure were monitored throughout (see Table 2).

**Table 2:** Anesthesia and procedural summary.

Procedure	Details
Premedication	Diazepam 0.2 mg/kg IV
Induction	Propofol 4 mg/kg IV to effect
Maintenance	Isoflurane (1.5–2.5%) in 100% oxygen via Dräger Fabius Plus
Positioning	Oblique right lateral recumbency, upper hindlimb secured cranially
Monitoring	End-tidal CO₂, SpO₂, ECG, esophageal temperature, non-invasive BP
Initial access	4 Fr urinary catheter advanced to bladder (patent penile urethra)
Diagnostic imaging	Retrograde urethrogram (iohexol) under fluoroscopy
Stricture location	Post-prostatic urethra (1 mm focal narrowing)
Urethrotomy	Dorsal internal urethrotomy using 1.3 × 130 mm microlancet (cold-knife)
Stent placement	Self-expanding nitinol stent (5.0 mm × 50 mm), laser-cut
Post-dilation support	14 Fr double-lumen balloon catheter, inflated with 5 mL metronidazole
Catheter management	Secured externally; daily owner-administered irrigation
Total procedure time	30 min
Intraoperative stability	No hypotension, hypothermia, or arrhythmias
Recovery	Uneventful; discharged same day
Postoperative medications	Vedaprofen 1 mg/kg PO SID; topical erythromycin; Elizabethan collar
Planned follow-up	Cystoscopy and catheter/stent evaluation at day 7 and 14

### 3.2 Diagnostic urethrography

**Figure 2:**
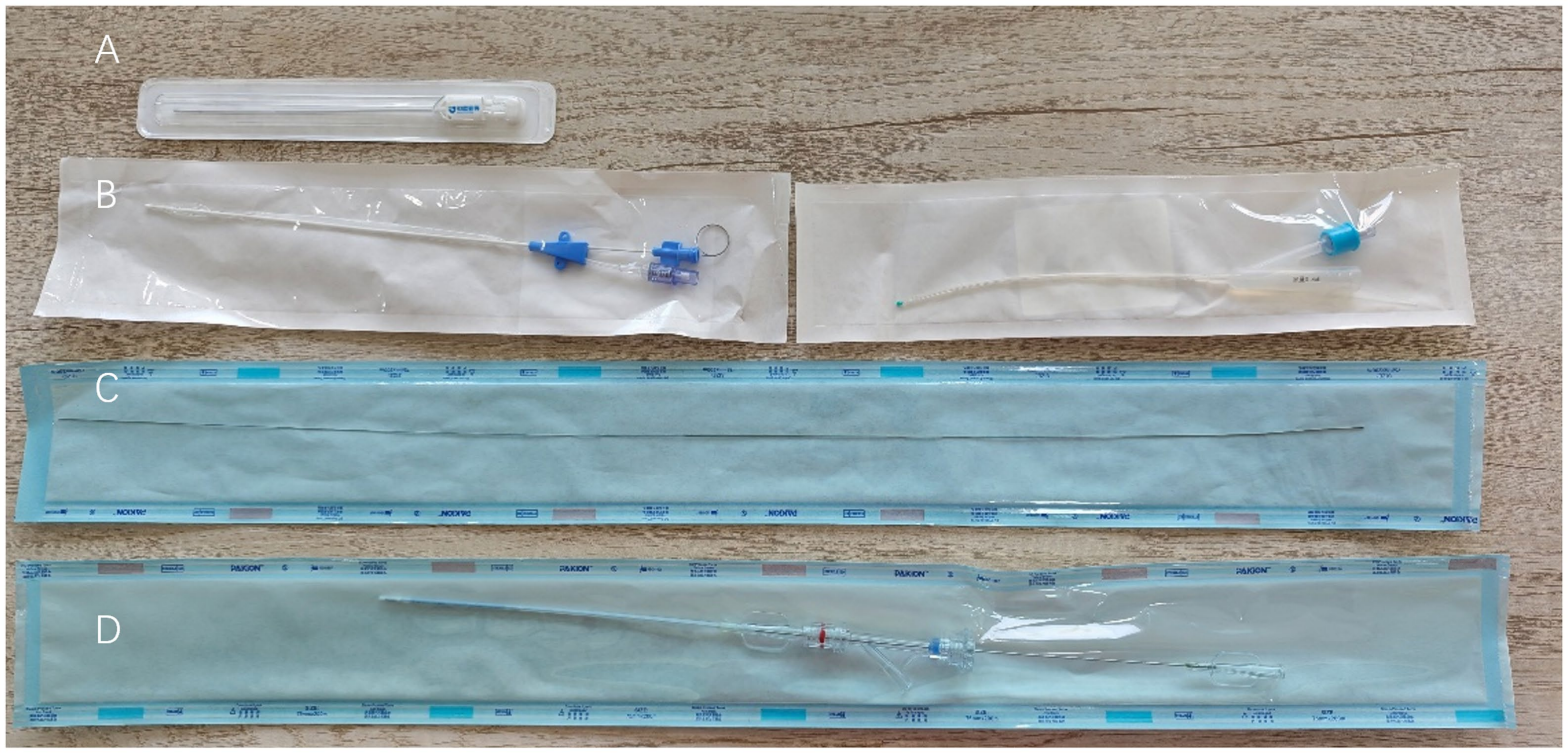
Instrumentation for the urethrotomy and stenting procedure. The components are arranged sequentially from top to bottom: **(A)** The urethral microlancet used for the internal incision, **(B)** Two standard urethral catheters, **(C)** The guide wire for instrument guidance, and **(D)** The laser-cut nitinol stent prior to deployment.

**Figure 3:**
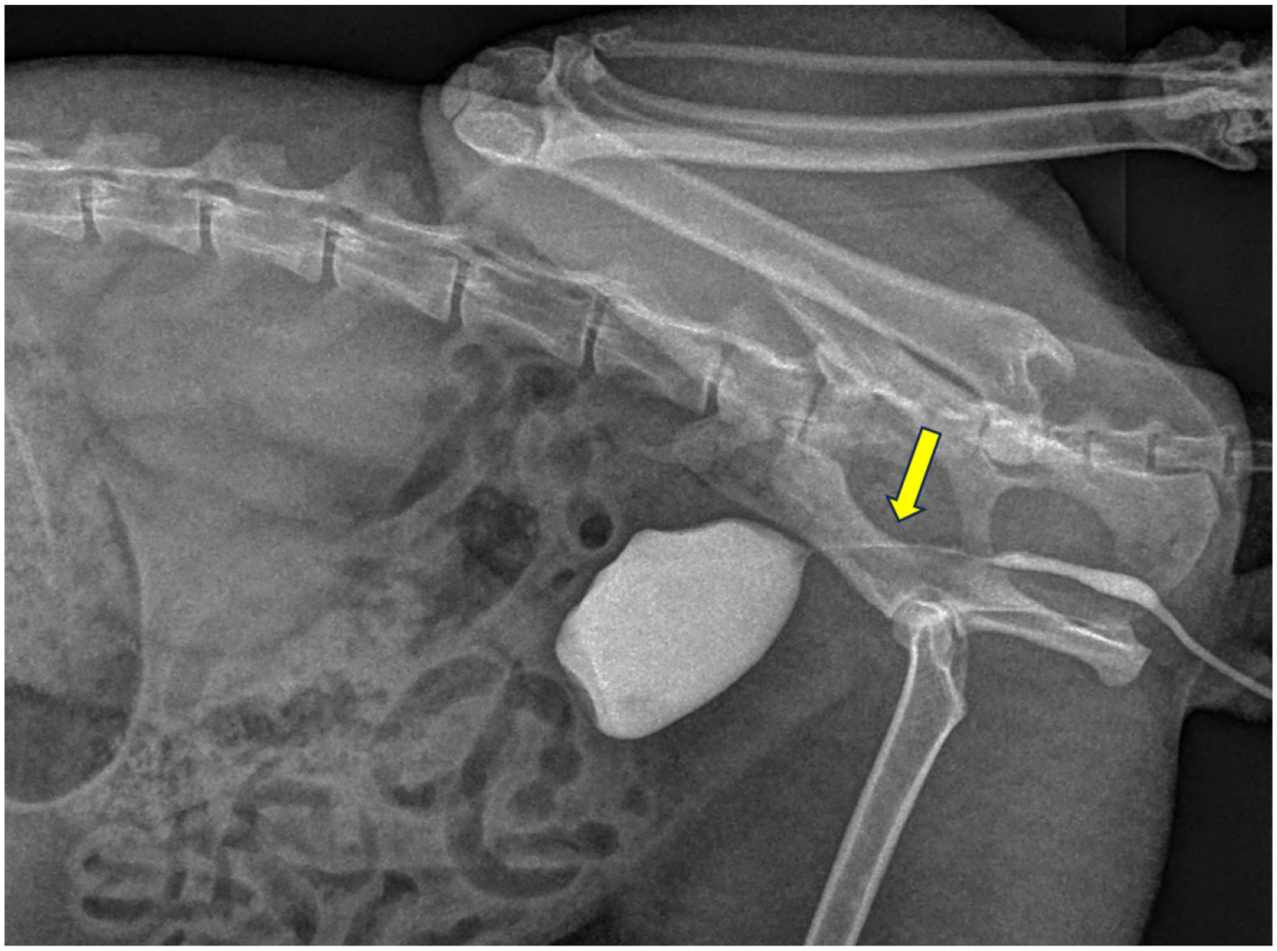
Fluoroscopic image showing 1 mm narrowing (arrow) at the post-prostatic urethra prior to urethrotomy.

A 5 Fr urinary catheter was easily advanced into the bladder, suggesting that the penile urethra was patent. The instruments used in this procedure are listed on Figure 2. A retrograde urethrogram was performed under fluoroscopy (Veterinary C-arm with flat-panel detector, Shanghai Wislong Pet Co. Ltd., China) using iohexol contrast delivered at the level of the proximal penile urethra, as shown in Figure 3. Total fluoroscopy time was 4.2 min with a DAP of 1.8 Gy·cm^2^; lead aprons and thyroid shields were used. Fluoroscopic imaging revealed a focal 1 mm stricture at the post-prostatic urethra extending cranially to the level of the bulbourethral glands. The contrast column terminated abruptly, and the bladder appeared compressed when distended.

### 3.3 Microlancet urethrotomy

**Figure 4:**
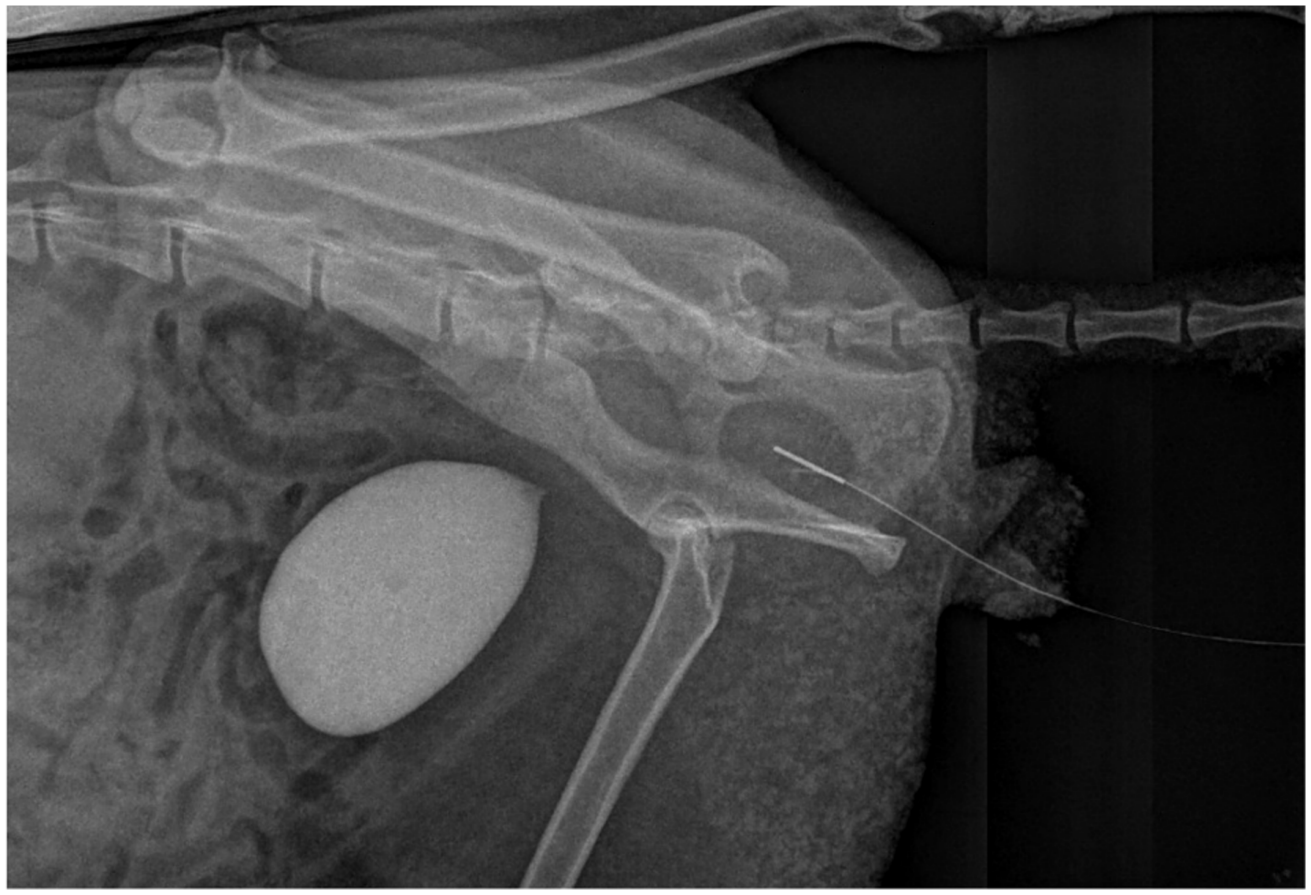
Urethral microlancet with ventral alignment marking used for internal incision.

The cat was placed in dorsal recumbency. A urethral microlancet (1.3 × 130 mm, Mingguang XinCui Biotechnology Co., LTD, China) was advanced retrograde through the urethra to the level of the bladder under fluoroscopic guidance. The black marker indicates the blade’s position, therefore, it was maintained facing upward throughout the procedure to ensure the blade remained oriented toward the patient ventral side prior to activation. Following manufacturer instructions, the handle was attached and the blade deployed internally. The microlancet was then withdrawn slowly and steadily to incise the ventral urethral mucosa from the stricture distally toward the penile urethra, as shown in Figure 4. Increased tactile resistance as the blade progressed distally indicated contact with the narrower penile urethra, signaling completion of the incision.

### 3.4 Stent deployment

**Figure 5:**
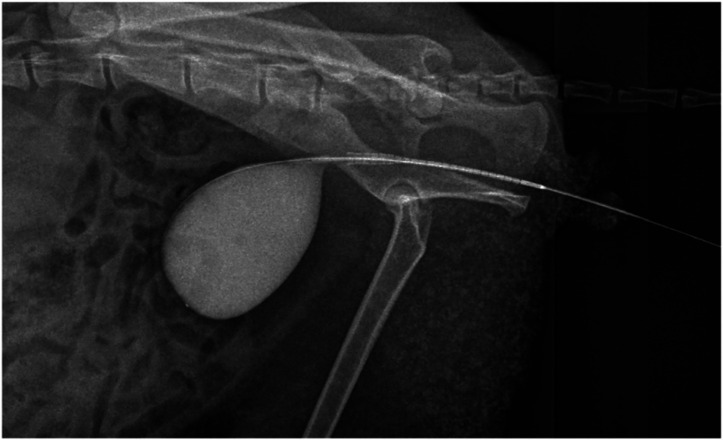
Stent deployment guided under flat-panel detector C-arm.

**Figure 6:**
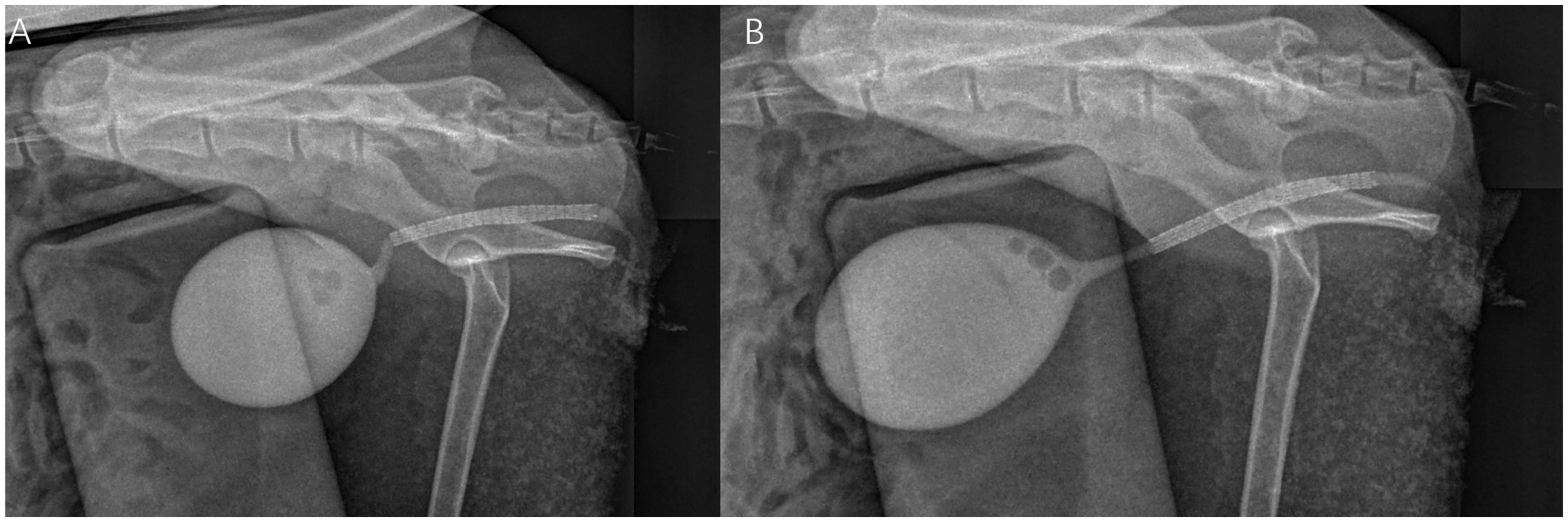
**(A)** Fluoroscopic image post-stenting showing restored luminal patency with nitinol stent *in situ*. **(B)** Manual bladder compression to simulate voiding pressure and confirm stent function under fluoroscopy.

Then a laser-cut self-expanding nitinol stent (5.0 mm diameter, 50 mm length, Figure 5, Mingguang XinCui Biotechnology Co., LTD, China) was then deployed across the stricture to scaffold the lumen. Then the deployment of the stent was checked by the C-arm, the post-stenting showing restored luminal patency with the nitinol stent *in situ* (Figure 6).

A 14 Fr double-lumen balloon catheter was inserted over the stent, inflated with 5 mL NSS, and secured externally. This provided urethral patency and minimized stent migration during the initial healing phase. 5 mL Metronidazole solution (25 mg) was infused into the urinary bladder. Total procedure time was under 30 min. No intraoperative hypotension, hypothermia, or arrhythmias occurred.

### 3.5 Postoperative care and follow-up

**Figure 7:**
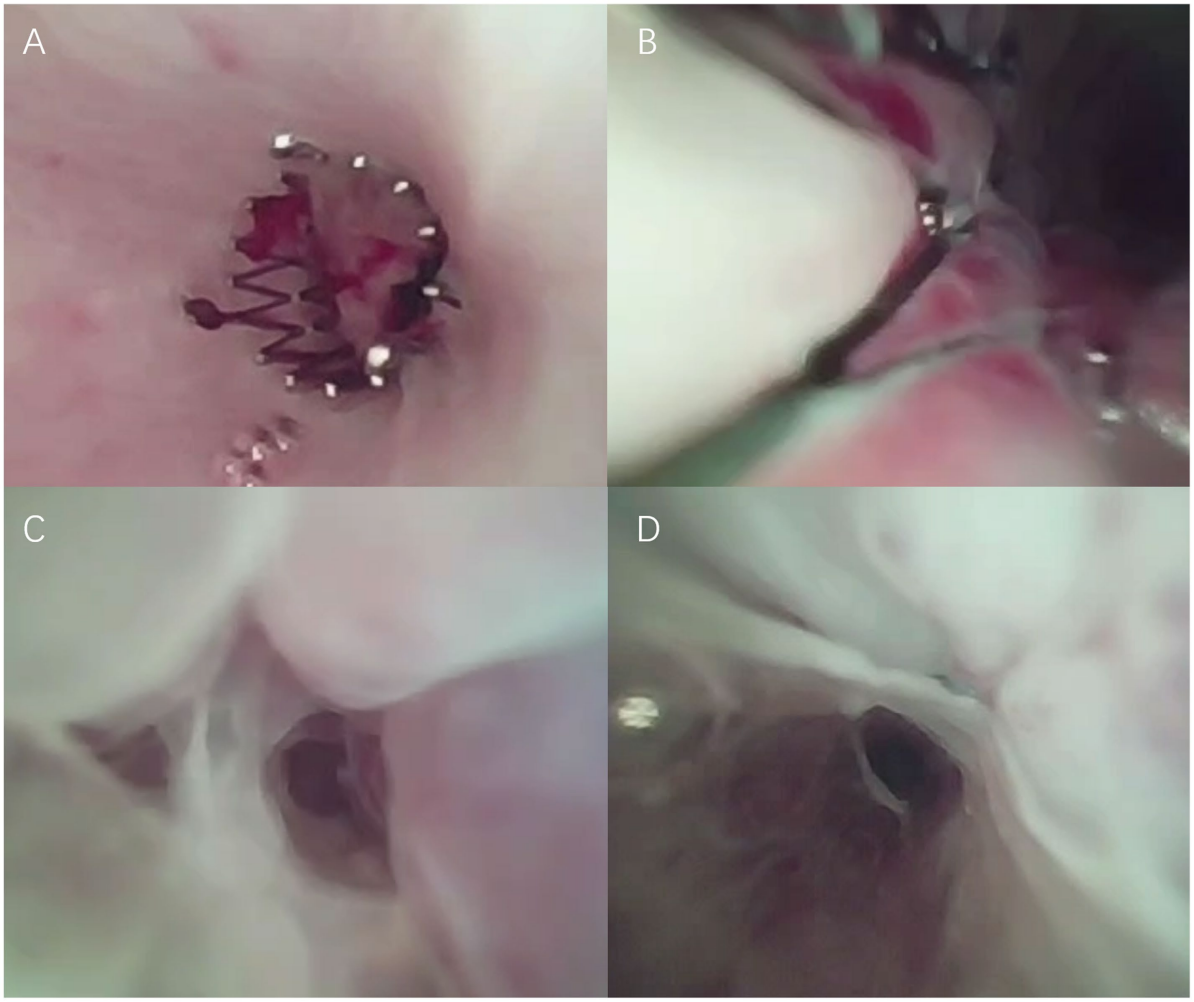
**(A)** Urethral stent observed under cystoscopy during anesthesia at 1 week post-operation. **(B)** Tissue growth beneath the urethral stent observed under cystoscopy at 1 week post-operation. **(C)** Urethral tissue growing into the stent observed under cystoscopy during anesthesia at 2 weeks post-operation. **(D)** Urethral tissue nearly encapsulating the stent observed under cystoscopy at 2 weeks post-operation.

Postoperatively, the cat recovered uneventfully and was discharged with an Elizabethan collar, oral anti-inflammatory therapy (Vedaprofen 1 mg/kg PO SID, Beijing Orbiepharm) for 5 days, and erythromycin ointment for local urethral care. The owners were instructed to irrigate the catheter daily with metronidazole solution and return for stent and catheter removal at day 14. The cat exhibited normal behavior at home. One week later, the owner consented to cystoscopy and associated evaluations to assess urethral healing and determine suitability for urinary catheter removal, as shown in Figure 7. Gently extrude the penis; clean the prepuce/penile tip with weak antiseptic and apply sterile lubricant. Connect warm sterile saline for continuous low-pressure irrigation to distend and visualize. Introduce the lubricated 1 mm scope directly into the penile urethral orifice under direct visualization. Advance slowly and gently through the penile, membranous, and pelvic urethra to the bladder neck, then enter the bladder while maintaining irrigation. Systematically inspect the urethral mucosa, bladder neck, trigone (ureteral openings), and bladder wall for abnormalities (e.g., inflammation, plugs, strictures, small calculi). The tissue growth beneath the urethral stent observed under cystoscopy at 1 week post-operation. Concurrent serum biochemistry and complete blood count (CBC) revealed no significant abnormalities, demonstrating marked improvement compared to prior results. At 14 days postoperatively, clinical and laboratory evaluations revealed no evident abnormalities. And cystoscopy under general anesthesia confirmed progressive urethral healing with early mucosal ingrowth beneath the stent; by 2 weeks, the epithelium had nearly encapsulated the nitinol mesh in Figures 7C,D. No adverse events such as balloon rupture or leakage were observed. Catheter retention lasted 14 days, after which the device was removed, while the stent remained *in situ*. The catheter were patent during the observation, and no major complications were observed. Cystoscopy examination at 7 and 14 days confirmed progressive mucosal ingrowth into the stent mesh and no evidence of granulation tissue or encrustation.

## 4 Discussion

This case illustrates the novel use of internal urethrotomy and metallic stenting to relieve an intrapelvic urethral stricture in a cat—a region inaccessible to standard perineal urethrostomy. Fluoroscopic retrograde urethrogram identified a 1 mm post-prostatic stricture, prompting endoluminal ventral microlancet incision, followed by nitinol stent placement and double-lumen catheter stabilization. To our knowledge, this is the first report describing this minimally invasive method in a feline patient. The technique enabled restoration of urinary patency without open surgery or the morbidity associated with urethrostomy. The approach combined human-derived principles adapted for feline anatomy and offers a promising endoluminal solution for proximal urethral strictures, a region that is traditionally challenging to access in small animals.

Traditional PU, although effective for distal urethral obstructions, cannot address strictures within the pelvic urethra (4). However, PU does not resolve obstructions located cranial to the bulbourethral glands. More invasive alternatives such as antepubic or transpelvic urethrostomies are technically feasible but carry high risks, including dehiscence, incontinence, peritonitis, and urine scald dermatitis. Balloon dilation, though less invasive, requires antegrade access to treat intrapelvic lesions, necessitating a surgical cystotomy in cats due to the narrow and curved urethral lumen, which prevents retrograde urethroscopy (10). Retrograde urethroscopy is not feasible due to the feline urethra’s narrow and curved anatomy; successful antegrade techniques require surgically exposing the bladder and accessing the urethra through a cystotomy (11, 12). This substantially increases procedural invasiveness, operative time, and risk.

In contrast, the microlancet technique enabled retrograde incision of the fibrotic segment under fluoroscopic guidance. Because retrograde endoscopy is not feasible in male cats, this approach allows a perineal-access procedure with direct visualization. The black alignment marker ensured that the cutting blade incised ventrally. This blade orientation, ventrally positioned was chosen deliberately to avoid damaging the dorsal prostatic region, which in dogs is rich in vascular parenchyma and at higher risk for hemorrhage. In the cat, the prostate does not fully encircle the urethra; the ventral aspect lacks glandular tissue and is instead composed primarily of urethralis muscle, making it a safer plane for incision. A continuous ventral incision was performed while withdrawing the blade from the bladder to the prepuce. Increased tactile resistance as the blade progressed distally indicated contact with the narrower penile urethra, signaling completion of the incision. This controlled maneuver allowed targeted expansion of the fibrotic stricture while minimizing collateral trauma.

The adaptability of this procedure to a fluoroscopic setting without cystotomy or rigid endoscopy represents a significant advancement. While PU and ballooning have clear roles (13), this endoluminal method offers a middle ground—providing stricture relief in anatomically challenging areas with lower surgical morbidity. Patient selection for this technique requires both anatomical and clinical consideration. Technically, the method is best suited for short (<1.5 cm) focal strictures located in the post-prostatic urethra. Clinically, it may benefit cats with concurrent comorbidities—such as HCM or polycystic kidney disease—who may not tolerate open abdominal procedures. The entire operation was completed in under 30 min, with minimal blood loss and no intraoperative complications, supporting the utility of this approach in high-risk patients.

A previous report indicate another case the maximum diameter of the dilated urethral segment cranial to the stricture was 5.5 mm (14, 15). Sizing followed canine urethral stenting guidelines, recommending stent diameter ≤ 1.3 times the maximum luminal diameter to achieve adequate mucosal apposition while reducing risks of migration or mucosal edema/inflammation (14, 15). In adult cats, prostatic urethral diameter averages 4.5–5.0 mm; the observed 5.5 mm likely represents compensatory dilation post-stricture (16). CT reference values in healthy cats show a mean total urethral thickness of 2.20 ± 0.26 mm (unaffected by sex, breed, weight, or bladder volume), compared with 2.75 ± 0.51 mm in cats with lower urinary tract signs (17). With self-expanding nitinol SEMS, progressive tissue ingrowth around struts is expected. Accounting for normal urethral thickness (around 2.2 mm), stent diameters of 5–6 mm allow sufficient residual space for incorporation without compromising patency or causing excessive compression. A 5 mm, 5.5 mm or 6 mm stent we believe were appropriate, optimizing stricture relief, anchorage, and accommodation of physiological tissue response in the feline urethra. Thus, we choose a 5 mm stent in this case.

However, limitations persist. The feline urethra’s narrow diameter leaves little margin for instrumentation error. Stents may promote granulation tissue, encrustation, or infection. Without long-term follow-up, the durability of this intervention remains speculative. Despite this, intraoperative success and restoration of urine flow demonstrate the technical viability of this method. Beneficial early mucosal encapsulation can progress to excessive granulation tissue growth through uncovered nitinol mesh, causing luminal narrowing or obstruction over months to years. As foreign bodies, stents promote biofilm, calcium-based mineral deposition, and urolith formation, especially in cats prone to concentrated urine and recurrent LUTD. Analogous ureteral stent complications include encrustation and debris accumulation, potentially leading to obstruction, dysuria, or hematuria. However, these complication were not observed 3.5 months postoperatively in this case.

Cyclic urethral stresses from voiding, movement, or straining may cause material fatigue and fracture over time, risking fragments, migration, or re-obstruction. While no fractures were seen in short-term feline urethral cases, this risk is extrapolated from dynamic sites and ureteral stents requiring replacement.

The novel technique described herein fills an important therapeutic gap by offering a minimally invasive alternative to surgical urethrostomy or open diversion for proximal urethral strictures. Compared to perineal urethrostomy (PU), which is effective for distal obstructions but cannot address intrapelvic lesions, the microlancet-stent method preserves native anatomy and avoids a perineal wound, beneficial in high-risk patients. Unlike balloon dilation, which carries a higher risk of restenosis without structural support, the use of a self-expanding stent provides sustained luminal patency. Open procedures such as prepubic urethrostomy or resection-anastomosis carry high morbidity and long-term complication rates, making this fluoroscopy-guided approach a promising, lower-morbidity option. However, long-term stent performance in feline patients remains to be validated, and careful case selection and monitoring are essential.

Nevertheless, this report represents a proof-of-concept based on a single case. The follow-up period of 3.5 months is enough to provide mid-term observation, but it limits inference about long-term stent stability, risk of encrustation, or stricture recurrence. Human studies suggest that nitinol stents can undergo epithelial overgrowth, fracture, or migration over time. Veterinary reports also caution against chronic stent use without routine surveillance. Ongoing monitoring with serial imaging and cystoscopic evaluation is needed to validate long-term efficacy.

The preoperative elevation in CPK (741 U/L) was likely multifactorial, potentially reflecting prolonged stranguria with repeated abdominal straining, transient myocardial stress associated with dynamic left ventricular outflow tract obstruction, and anorexia, all of which are recognized contributors to increased CPK activity in cats. The rapid normalization of CPK following relief of urinary obstruction and resumption of food intake supports a reversible, stress-related etiology, despite persistence of the underlying cardiac disease. Preoperative hyperglycemia was similarly considered stress-induced and resolved without intervention. No direct association with the urethral stricture or renal cysts was identified. And we believed that this stent and microlancet procedure provided better care for this cat. At the time of writing (3.5 months postoperatively), the cat remains clinically normal with no recurrence of clinical signs.

This report highlights a promising, minimally invasive technique for treating proximal urethral strictures in cats. It provides a proof-of-concept for fluoroscopic-guided retrograde urethrotomy with stenting, eliminating the need for invasive antegrade or open approaches. Broader clinical application will require further cases, optimization of stent design for feline anatomy, and refinement of postoperative care protocols. Notably, this technique’s ability to incise the entire urethra in one motion may have potential applications in feline idiopathic cystitis (FIC) cases with distal urethral obstruction, though further investigation is warranted to assess safety and efficacy in that context.

## 5 Conclusion

This report describes the first documented use of a urethral microlancet for ventral internal urethrotomy combined with nitinol stent placement in a cat with an intrapelvic urethral stricture. The technique achieved rapid restoration of urethral patency and avoided the need for extensive surgery. The intraoperative and immediate postoperative results suggest the feasibility of this minimally invasive method. This approach may represent a useful alternative to perineal urethrostomy or prepubic urethrostomy in anatomically unfavorable feline strictures. Further clinical cases and follow-up studies are needed to evaluate the safety, efficacy, and long-term outcomes of this novel technique in feline patients.

## Data Availability

The data supporting the findings of this case report, including clinical history, laboratory results (hematology and biochemistry), procedural details, and all imaging (echocardiography, ultrasonography, fluoroscopy, and cystoscopy), are presented within the article in the form of tables, figures, and descriptive text. Requests to access the datasets should be directed to DM, danfuma@njau.edu.cn.
