# Supplementary material for: Microlancet-assisted internal urethrotomy with stent placement for feline pelvic urethral stricture: a case report
Source: Front Vet Sci. 2026 Mar 25;13:1755771. doi: 10.3389/fvets.2026.1755771 (PMC13056664; doi:10.3389/fvets.2026.1755771)
Supplement: Supplementary file 1 [file Image_1.pdf]

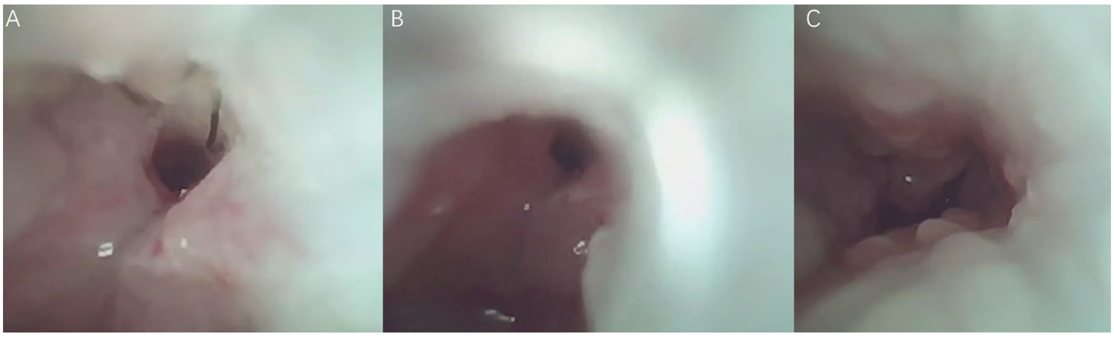

Figure S1. A. Urethral stent observed under cystoscopy during anesthesia at 3.5 months post operation. B. Tissue growth around the urethral stent observed under cystoscopy at 3.5 months post operation. C. Tissue ingrowth with visible intraurethral hyperplasia, while the urethra remains patent.
